# Assessment of micro RNAs expression in leukemic cells as prognostic markers in chronic lymphocytic leukemia: micro RNAs can predict survival in a course of the disease

**DOI:** 10.18632/oncotarget.24927

**Published:** 2018-04-10

**Authors:** Agnieszka Szymczyk, Sylwia Chocholska, Arkadiusz Macheta, Dariusz Szczepanek, Marek Hus, Monika Podhorecka

**Affiliations:** ^1^ Department of Haematooncology and Bone Marrow Transplantation, Medical University of Lublin, Lublin, Poland; ^2^ Independent Clinical Transplantology Unit, Medical University of Lublin, Poland; ^3^ Department of Neurosurgery and Pediatric Neurosurgery, Medical University of Lublin, Lublin, Poland

**Keywords:** chronic lymphocytic leukemia, micro RNA, overall survival, prognostic factors, progression free survival

## Abstract

Numerous genetic alterations predicting prognosis and clinical outcome are revealed recently in chronic lymphocytic leukemia (CLL). Among them the deregulated expression of micro RNAs that can induce tumor growth, or act as tumor suppressors seem to be of great importance. This study aimed to analyze the possible role of chosen micro RNAs as markers of prognosis in patients with CLL. We assessed the expression of miR-21, miR-34a, miR-181a, miR-199a/b and miR-221 in previously separated leukemic cells with the use of qRQ-PCR technique at the moment of diagnosis. The results were then analyzed in regards to presence of prognostic factors, clinical data and the end points like progression free survival (PFS), time to progression (TP) and overall survival time (OS).

We detected significant correlations between expression of the analyzed micro RNAs and CLL prognostic markers particularly as far as miR-221 and miR-181a were concerned. The subsequent analysis revealed that high expression of miR-34a and miR-181a as well as low miR-21 expression indicated longer TTP, while miR-221 was predictor of OS.

The obtained results prove the role of micro RNAs as CLL prognostic markers, particularly as factors predicting survival in a course of the disease.

## INTRODUCTION

Chronic lymphocytic leukemia (CLL), one of the most frequently diagnosed leukemias, is characterized by the accumulation of leukemic CD19+/CD5+/CD23+ B cells in the blood, bone marrow, lymph nodes and spleen [1, 2]. Clinical course and prognosis of this type of leukemia are highly variable. Some patients with benign disease never require therapy and die because of causes other than leukemia. In others the treatment is started soon after diagnosis, because of the aggressiveness of the disease [3, 4]. Numerous factors are used to predict prognosis and clinical outcome of CLL patients like the mutational status of immunoglobulin heavy chain genes (IgVH)), ZAP-70 and CD38 expression as indicators for IgVH mutations as well as gene and genomic abnormalities [5, 6, 7].

Lots of mechanisms involved in leukemic transformation of CLL are reported. The B cell receptor signaling plays an important pathogenic role because of BCR-dependent survival of leukemic lymphocytes [8, 9]. Pro-proliferative signals are also mediated from microenvironment composed of macrophages, T cells, or stromal follicular dendritic cells. This microenvironment produces various essential proteins like chemokines and cytokines that by interacting with leukemic cells may induce their survival [7, 10, 11]. Additionally, numerous genetic alterations are revealed recently in CLL. These are single- nucleotide polymorphisms, chromosomal alterations and alterations in non-coding RNA, like micro RNA (miR) [7, 9, 12]. Micro RNAs belong to epigenetic regulators that modulate gene expression and cellular signaling pathways. Micro RNAs may be deregulated in human cancers, some of them induce tumor growth, while others act as tumor suppressors [13, 14]. They expression can be used to predict prognosis and clinical response to treatment in cancer patients [13, 15]. CLL was the first proliferative disorder that was reported to be connected with alterations in micro RNAs. In particular, miR-15a and mirR-16-1 both target BCL2 and MCL1 expression are dysfunctional in about 60% of patients with CLL [7, 16, 17]. Such abnormalities lead to the resistance of B lymphocytes towards apoptosis. Attention has also focused on other micro RNAs in CLL patients that are dysregulated and may be overexpressed or show low level. Thus extensive research are currently conducting to find the pattern of micro RNA expression in CLL patients which could be used as prognostic factor in everyday clinical practice.

The presented article aimed to analyze the possible role of chosen micro RNAs as a markers of prognosis in patients with CLL. The expression of miR-21, miR-34a, miR-181a, miR-199a/b and miR-221 in previously separated CD19+ leukemic cells was assessed with use of qRQ-PCR technique at the moment of diagnosis. The obtained results were then analyzed in regards to presence of prognostic factors, clinical data and the end points like progression free survival (PFS), time to progression (TP) and overall survival time (OS).

## RESULTS

### Assessment of micro RNAs expression in leukemic cells

Our analysis revealed an expression of all analyzed micro RNAs in leukemic cells except of miR-199a/b, so this micro RNA was not further assessed. Micro RNAs expression presented as mean ± standard deviation are shown in Table 1. According to the level of particular micro RNA expression the patients were divided into groups of low and high expression, respectively (see Table 1). The cut-off point was established as the mean expression of micro RNA in the study group.

**Table 1:** Micro RNAs expression presented as mean ± standard deviation (M ± SD)

Examined microRNA	microRNA expression		
	Whole study group M ± SD (*n*)	Low expression M ± SD (*n*)	High expression M ± SD (*n*)
**miR-21**	0.064 ± 0.061 (40)	0.026 ± 0.022 (22)	0.111 ± 0.061 (18)
**miR-34a**	0.041 ± 0.074 (40)	0.010 ± 0.011 (29)	0.122 ± 0.105 (11)
**miR-181a**	0.028 ± 0087 (40)	0.001 ± 0.004 (30)	0.111 ± 0.151 (10)
**miR-199a/b**	0.000 ± 0.000 (40)	NA	NA
**miR-221**	0.045 ± 0.078 (40)	0.016 ± 0,014 (30)	0.131 ± 0.122 (10)

According to level of particular micro RNA expression the patients were divided into groups of low and high expression, respectively. The cut-off point was assumed to be the mean expression of a micro RNA in the study group. NA-not applicable, *n*-number of patients.

### Assessment of micro RNAs expression in leukemic cells in relation to CLL prognostic markers

**Figure 1:**
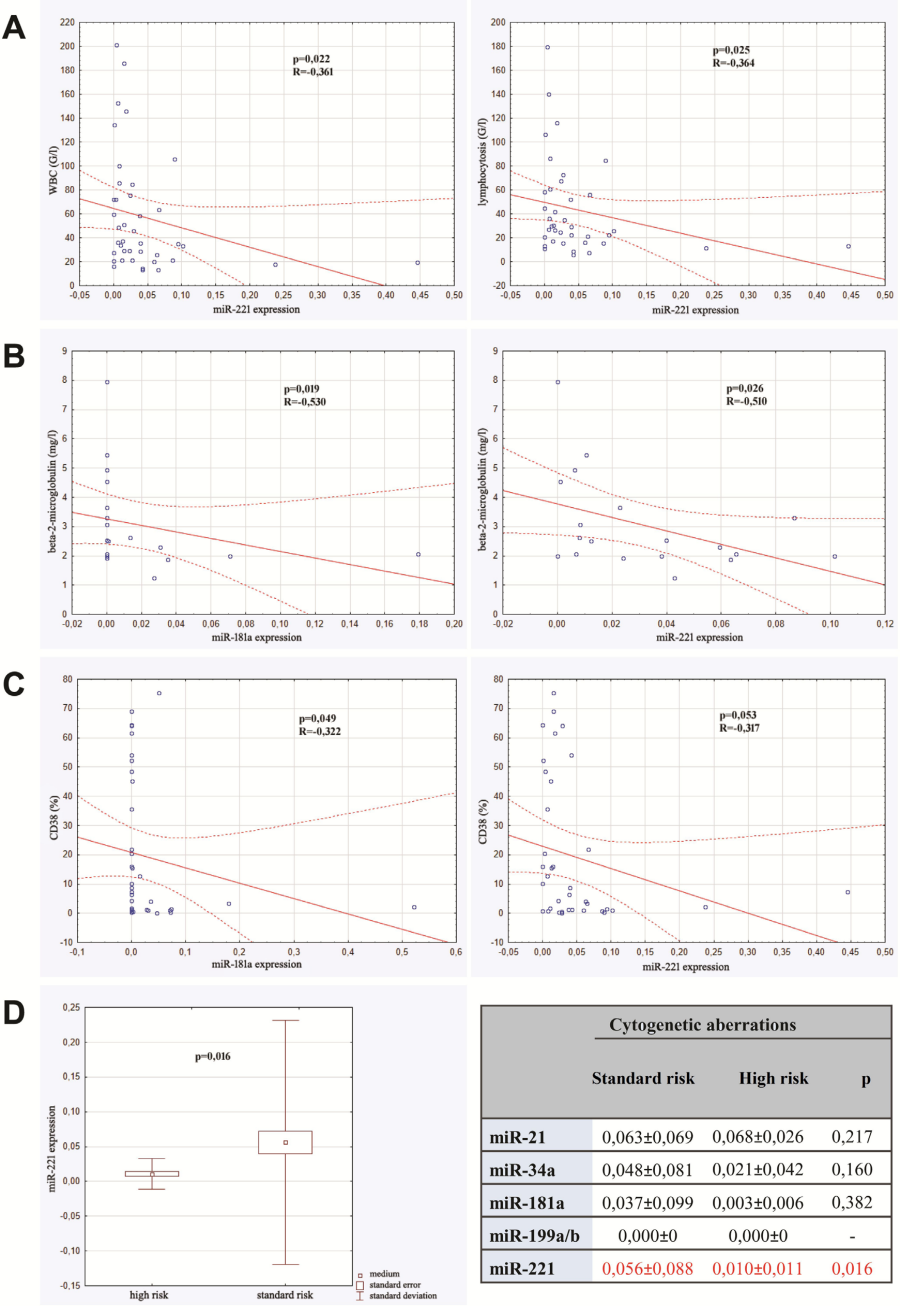
Correlations between micro RNAs expression and prognostic factors of CLL **(A)** Correlation between miR-221 expression and both leucocytosis and lymphocytosis. **(B)** Correlation between miR-181a expression and of β2-mikroglobulin level and between miR-221 expression and β2-mikroglobulin level. **(C)** Correlation between miR-181a expression and CD38 expression and between miR-221 expression and CD38 expression. **(D)** Micro RNAs expression in high risk cytogenetic patients versus standard risk group.

We detected statistically significant negative correlation between expression of miR-221 and both leukocytosis and lymphocytosis. Other micro RNAs were not in correlation with parameters of complete blood count. The expression of both miR -181a and miR-221 were in adverse significant correlation with serum level of β2-mikroglobulin. These micro RNAs were also in negative correlation with expression of CD38 on leukemic cells, however such a relationship was not found as far as expression of ZAP-70 was concerned. Additionally, expression of miR-221 was detected to be different in regard to cytogenetic risk. In a group of standard-cytogenetic risk the expression was higher in comparison to high-risk group. The results are shown in Figure 1. We did not detect any correlation between expression of miR-21, miR-34a, miR-181a, miR-221 and age and sex of the studied patients. There was no relationship between micro RNA expression and clinical stadium of CLL, either.

### Assessment of influence of microRNA expression on overall response rate

The group of studied patients was assessed in regards to first line chemotherapy regimens as well as chemotherapy outcome. Only the subjects (20 persons) who required therapy because of the disease progression were enrolled into this part of study. The following schemes of chemotherapy were used: chlorambucil + prednisone (9 persons), fludarabine + cyclophosphamide (7 persons), fludarabine + cyclophosphamide + rituximab (2 persons), bendamustine (1 person), cyclofosfamide + vinkristin + doksorubicin + prednisone (1 person). The patients were classified as responders (those who obtain complete or partial remission) and non-responders (those who have stable disease or progression). Statistical analysis indicated that expression of analyzed micro RNAs was not a predictor of chemotherapy outcome, however the variability of regimens used and a low number of patients in each group might influence the results.

### Analysis of microRNA expression in regard to time to progression

**Figure 2:**
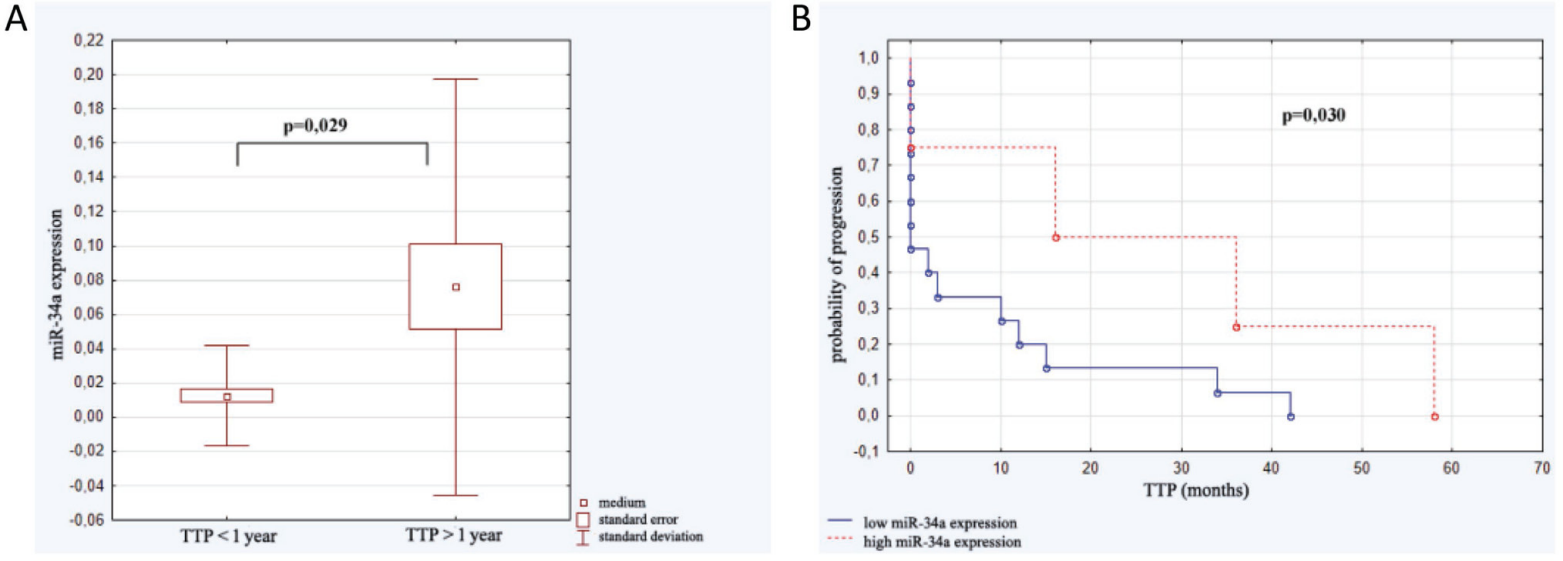
Expression of miR-34a in relation to time to progression (TTP) **(A)** miR-34a level in patients with moment of progression which occurred within 1 year after diagnosis (*n =* 14) and in subjects with time to progression longer than 1 year (*n =* 6). **(B)** Kaplan–Meier analysis of progression probability in a group of patients with high versus low expression of miR-34a.

**Figure 3:**
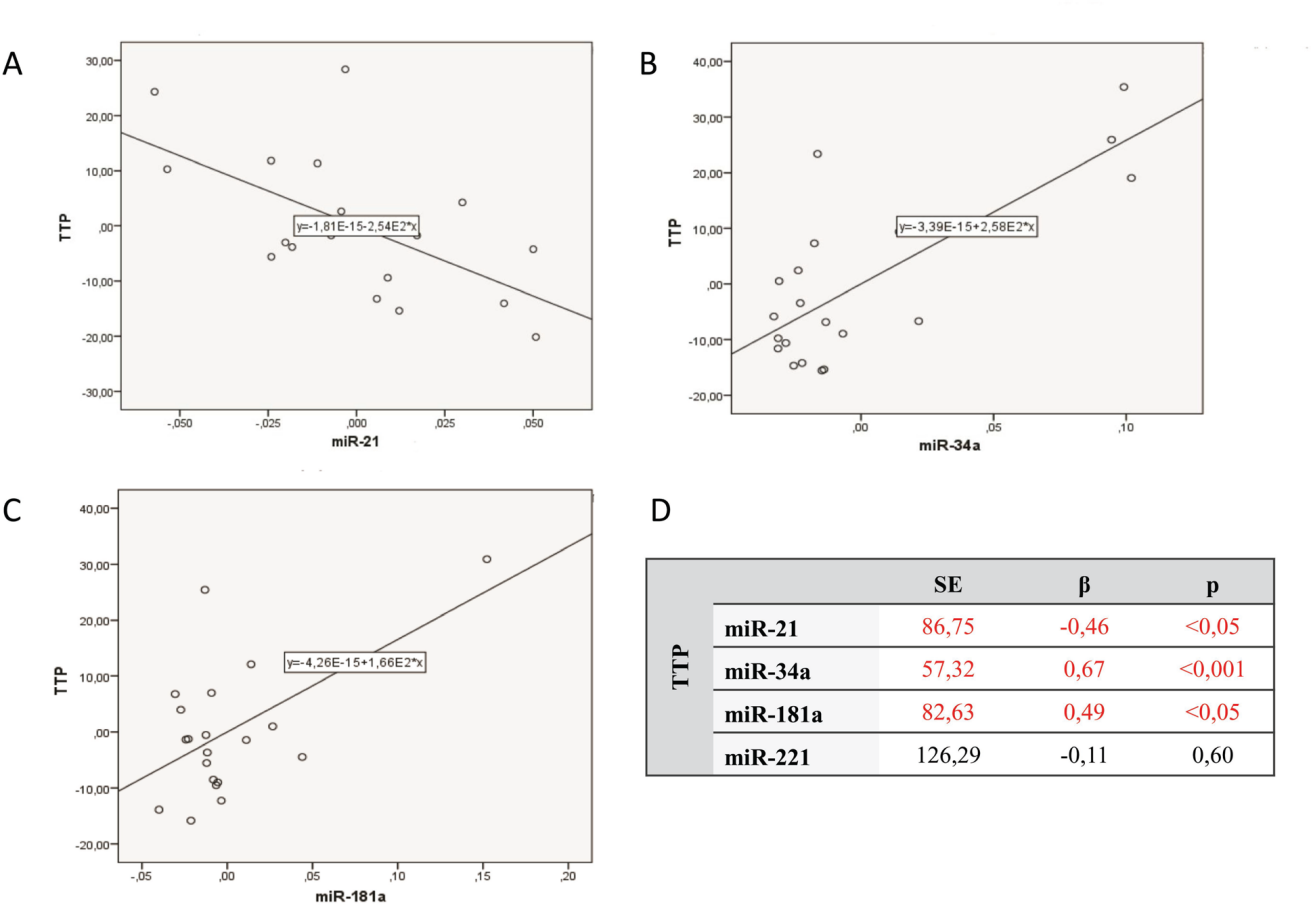
Multivariate linear regression analysis assessing influence of all studied micro RNAs on time to progression (TTP) **(A)** Expression of miR-21 versus TTP. **(B)** Expression of miR-34a versus TTP. **(C)** Expression of miR-181a versus TTP. **(D)** Data of numerical analysis.

The study group was analyzed with reference to time to leukemia progression. In the moment of analysis 20 out of 40 enrolled patients were diagnosed because of leukemia progression. The others were in stable phase of the disease. The patients with progression detected were divided into 2 groups: the first one with moment of progression which occurred within 1 year after diagnosis (*n =* 14; 1.93 ± 3.97 months ) and the second one with time to progression longer than 1 year after beginning of leukemia (*n =* 6; 33.50 ± 16.29 months). Analysis of micro RNAs expression in the above groups revealed the statistically significant difference in expression of miR-34a. Similarly, Kaplan–Meier analysis of progression probability showed statistically significant difference between a group of patients with high versus low expression of miR-34a. These results are shown in Figure 2. As far as expression of miR-221, miR-21, miR-181a were concerned no statistically significant differences were detected. However, the multivariate assessment based on linear regression model assessing influence of all studied micro RNAs on TTP revealed statistical significance (*F =* 7.79; *p <* 0,001; ∆R^2^ = 0.59). High expression of miR-34a (β = 0.67) and miR-181a (β = 0.49) as well as low miR-21 expression (β = –0.46) significantly influence TTP. The data are presented in Figure 3.

### Assessment of influence of microRNA expression on progression free survival

The analysis of PFS was performed in the group of subjects who have completed first-line therapy (20 persons). They were divided into two groups: the first one of patients who progressed after the first line of treatment within one year (*n =* 12; M ± SD = 2.0 ± 3.232 months) and the second one who progressed in the period longer than one year (*n =* 8; M ± SD = 27.0 ± 11.402 months). Analysis of microRNA in the above groups revealed statistically significant difference only in case of miRNA-221 expression (0.016 ± 0.028 versus 0.031 ± 0.021, *p* = 0.045). Analysis based on linear regression in regards to all studied micro RNAs showed no statistical significance (*F =* 0.23; *p <* 0.92; ∆R^2^ = –0.24). Assessment with Kaplan–Meier test showed no statistically significant difference in predicting PFS between groups with high and low micro RNA expression, respectively.

### Assessment of microRNA expression as predictor of overall survival

**Figure 4:**
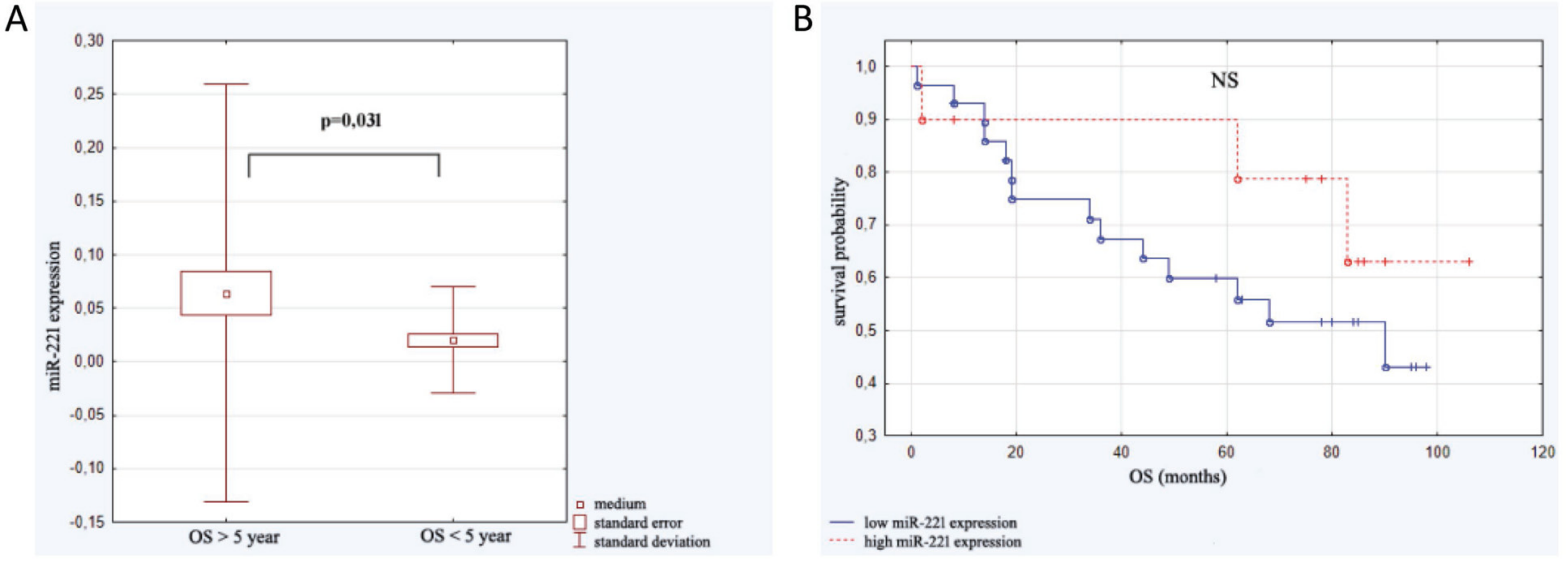
Expression of miR-221 in relation to overall survival (OS) **(A)** miR-221 level in patients who survived less than 5 years (*n =* 16) and those who survived more than 5 years (*n =* 24). **(B)** Kaplan–Meier analysis of progression probability in a group of patients with high versus low expression of miR-221.

The length of patient observation was between 1 and 106 months (59.90 ± 33.88). Up to the moment of analysis 45.2% of patients (*n =* 19) died. All subjects were divided into two groups: the first one - patients who survived less than 5 years (*n =* 16; 21.87 ± 17.23 months ) and the second one -those who survived more than 5 years (*n =* 24; 84.24 ± 12.08 months). Analysis of micro RNAs in the above groups revealed statistically significant difference in miRNA-221 expression. The linear regression assessment did not revealed statistical significance (*F =* 1.31; *p <* 0.29; ∆R^2^ = 0.03). Analysis with Kaplan–Meier test showed that the probability of survival was higher in the group of patients with elevated level of miR-221 expression in comparison to the group with lower miR-221 level, however the difference was not statistically significant (Figure 4).

### Influence of micro RNAs expression on patients morbidity

Until the moment of investigation 19 persons of the study group died. With a use of linear regression analysis we assessed correlation between miR-21, miR-34a, miR-181a and miR-221 expression and risk of death. However no statistically significant differences were found.

## DISCUSSION

Micro RNAs seem to be very informative biomarkers of clinical value that are easy-to-assessed both in cells and in body fluids [18, 19]. The role of particular micro RNAs expression in CLL patients was investigated and reported, however the one micro RNAs profile for this disease was not precisely established yet. Thus we tried to assess the role of the expression of following micro RNAs: miR-21, miR-34a, miR-181a, miR-199a/b and miR-221 as prognostic factors in CLL in relation to course of the disease and survival of patients.

Firstly, the correlation of micro RNAs expression and the established CLL prognostic factors was analyzed. We detected that low expression of miR-221 was observed together with higher leucocytes and lymphocytes concentration that may indirectly indicates the higher rate of leukemic cells proliferation. In the group of subjects with high cytogenetic risk (del 17p or del11q) miR-221 expression was significantly lower than in those with standard cytogenetic risk. Additionally, we detected negative correlation between miR-221 expression and β-2-mikroglobulin level. It can be concluded that a low expression of this micro RNA results in more progressive course of leukemia and is correlated with the presence of high-risk-cytogenetic profile. In case of miR-181a the correlation with CD38 expression was detected. In patients with low miR-181 level the expression of CD38 was significantly higher. Similarly, in patients with higher β-2-mikroglobulin concentration the expression of miR-181a was significantly lower. There were no detectable difference between expression of other micro RNAs and prognostic factors. Expression of miR199a/b was not detected at any of studied patients indicating that this miRNA cannot be consider as prognostic factor in CLL.

The literature data on micro RNA expression in course of CLL are incomplete and sometimes confused. Rodrigez *et al.* [20] showed that miR-221 is significantly lower in patients with del 17p, del11q and del13q in comparison to other patients. Contrary, Callin *et al.* [21] indicated the high expression of this micro RNA in cases with poor cytogenetic risk and aggressive course of leukemia. MiR-199a is widely reported to be changed in tumors like ovarian cancer, thyroid gland cancer and breast cancer [22–25]. Tropan *et al.* [26] assessed expression of this micro RNA in patients with DLBCL and indicted differences between cases with central nervous system infiltration and those without such manifestation. Pallash *et al.* [27] showed low expression of miR-199b in course of CLL, similarly to our results. Visone *et al.* [28] analyzed some micro RNAs expression in CLL patients of different cytogenetic risk groups. They indicated that in newly diagnosed subjects the presence of del 17p, high ZAP-70 expression and unmutated IgVH correlated with low expression of miR-29b, miR-29c, miR-223 and miR181 which indicated the shorter TTP. However, overexpression of miR-181a co-existing with trisomy 12 significantly downgraded prognosis [28]. The role of miR-34a expression in CLL course was previously examined as well. Its low expression was proved to be in correlation with del 17p and/or TP53 mutation. Additionally, abnormal miR-34a expression influenced expression of genes involving in CLL pathogenesis TCL1, BCL2, MCL1 as well as cykline D1 and p21 [29]. Similarly, miR-181a expression was demonstrated as regulator of TCL1 oncogene. In the advanced and aggressive stadium of leukemia miR-181a expression was significantly decreased [30]. Rossi *et al.* [31] indicated that in CLL patients with del 17p the overexpression of miR-21, miR-155, miR-15atogether with low miR-34a and miR-181b expression are characteristic. Additionally, the authors proved the correlation between the level of TP53 abnormalities and miR-34a and miR-155 expression [31].

Our analysis of micro RNAs expression in regards to end points in CLL patients revealed that high expression of miR-34a and miR-21 as well as low miR-181a expression significantly influenced TTP, while miR-221 and miR-181a indicated OS. Some literature data showed correlations of microRNA expression and the end points in hematologic disorders. Marcuccii *et al.* [32] indicated in group of patients with acute myeloid leukemia, that high miR-181a expression is connected with higher rate of complete remission and longer OS [32]. In DLBCL patients low level of miR-181a expression correlated with shorter OS [33]. Zhu *et al.* [34] assessing CLL patients showed correlation between low miR-181a expression and survival time. Analysis of miR-21 expression showed the correlation of its high level and poor prognosis in liver cancer, colon cancer and osteosarcoma [35–37]. There is also evidence on the correlation between high miR-21 expression and both OS and PFS in DLBCL patients [38, 39], however different results indicating that high miR-21 expression was in correlation with poor prognosis in this group of patients were also reported [40]. In regards to miR-221 expression there are results showing the correlation between its overexpression and shorter OS in liver cancer and non-small cell lung cancer [41, 42]. There is no evidence on miR-221 expression in CLL, however the correlation between its level and poor cytogenetic profile was reported. Thus, it may be presumed that this micro RNA overexpression will be observed together with shorter OS and poorer prognosis [20, 21].

The group of studied patients was assessed in regards to chemotherapy outcome, however no significant correlations were detected. Scientific literature data indicated that expression of specific micro RNAs may help to predict the chemotherapy outcome in CLL patients. Moussay *et al.* [43] showed the correlation of high miR-29, low miR-181 and low miR-221 expression with good response to fludarabine-based therapy. Similarly, Zhu *et al.* [44] proved the connection of fludarabine sensitivity and low miR-181 expression. Contrary, in patients with resistance to fludarabine the mir-21 overexpression and low expression of miR-34a were reported [45, 46]. In our study the variability of regimens used and a low number of patients in each group might influence the results. Thus further assessment in the bigger group of patients will be continued.

To summarize, based on our results and the presented literature data we can conclude that micro RNAs may be the prognostic factors in the course of the disease and the predictors for end points in CLL patients. Expression of miR-21, miR-34a and miR-181a may be useful in predicting TTP, while expression of miR-221 may indicate OS. Thus further studies are required in this field of CLL biology to prove the importance of micro RNAs expression in clinical practice.

## MATERIALS AND METHODS

### Research material

Forty newly diagnosed CLL patients, previously not treated, were enrolled into the study. The diagnosis of CLL was based on clinical examination and morphological and immunological criteria. The clinical characteristics are shown in Table 2. The local Bioethics Committee granted permission to conduct the research and patients were asked to sign the informed consents. Samples of peripheral blood were collected in syringes with the anticoagulant edetate (Sarstedt, Germany).

**Table 2:** Clinical characteristics of analyzed patients

Number of patients			40	
**Age**	medium (years)		64.0	
	minimum - maximum		38–85	
**Sex**	women		22	
	men		18	
**Rai clinical stage**	early (0-I)		20	
	advanced (II-IV)		20	
**CD38 expression (cutoff point 30%)**	positive		10	
	negative		30	
**ZAP-70 expression (cutoff point 20%)**	positive		12	
	negative		28	
**Cytogenetic aberrations**	low risk	del13q	13	30
		trisomy 12	3	
		normal	15	
	high risk	del 11q	7	10
		del17p	3	
**Lymphocytosis (G/l)**	medium		42,37	
	minimum - maximum		5,76–179,4	
**β**_2_**-microglobulin plasma level (mg/l)**	medium		3,12	
	minimum - maximum		0,19–7,96	
**Lactate dehydrogenase activity (U/l)**	medium		404,75	
	minimum - maximum		256–897	

### Isolation of B-cells from peripheral blood mononuclear cells with MACS method

Peripheral blood mononuclear cells (PBMC) were separated by density gradient centrifugation (Biocoll AG Biochrom, Germany). After washing with phosphate-buffered saline, the number and viability of cells were assessed with trypan blue staining. Viability below 95% was a disqualifying criterion for further study. PBMC were subsequently subjected to the procedure of leukemic cells separation. The magnetic activated cell sorting method was used according to manufacturer’s instruction (MACS Cell Separation, Germany). PBMC were firstly incubated with magnetic beads coated with monoclonal antibody (MoAb) anti-CD19 in MACS buffer and then subjected to washing procedures to obtain the cell pellet for application to the MACS column. In this way the CD19+ leukemic cells were obtained, their viability was assessed and they were used for further procedures.

### RNA extraction

Total RNA was isolated from leukemic cells according to the modified method of Chomczynski and Sacchi [47]. The mirVana Isolation Kit (Ambion, USA) for obtaining RNA was used. RNA concentration and integrity was determined by spectrophotometer. Isolated RNA was stored at –20° C until further procedures.

### MicroRNA quantification

Assessment of microRNA expression was performed using the TaqMan^®^ Small RNA Assays Kit (Applied Biosystems, USA). Initially, total cellular RNA was reverse-transcribed with specific primers using TaqMan^®^MicroRNA Reverse Transcription Kit according to the manufacturer’s protocol on Applied Biosystems 7500 Fast Real Time PCR Systems. The obtained complementary DNA (cDNA) was used for further procedures. The quantitative analysis of microRNA expression was done with quantitative reverse transcriptase real-time PCR, (qRT-PCR) method. Following probes were used: hsa-miR-21-5p, hsa-miR-34a-5p, hsa-miR-181a-5p, hsa-miR-199a-5p, hsa-miR-221-3p and hsa-miR-16 as endogenous control. A positive reaction was detected by accumulation of a fluorescent signal. The cycle threshold (Ct) was defined as the number of cycles required for the fluorescent signal to cross the threshold and exceeds background level. Ct levels were inversely proportional to the amount of microRNA in the sample. The expression of each micro RNA was normalized with the endogenous control. The comparative ΔΔCq method was then applied for data analysis, and fold changes were next calculated using 2^–ΔΔCq^ [48]. All PCR reactions were run in duplicate.

### Analysis of ZAP-70 and CD38 expression

Sample of PBMC cells were stained with the MoAbs anti-CD19 PE-Cy7, CD5 APC or CD3 PE (BD Pharmingen). After membrane staining, the cells were fixed by 1% paraformaldehyde solution. Next, anti-ZAP-70 antibody (Biomol Research Laboratories, USA) that was labeled by the ZenonTM Alexa Fluor^®^ 488 Mouse IgG2a Labeling Kit (Molecular Probes, USA) was added to the sample tubes. The samples were incubated for 30 min, washed, and examined by flow cytometry method. When ZAP-70 expression was detected in ≥ 20% of leukemic cells, the subject was considered positive for ZAP-70. To assess CD38 expression, PBMC were stained with anti-CD38 FITC, anti-CD19 PE, anti-CD5 CyChrome MoAbs, or IgG1 isotypic control (BD Pharmingen) for flow cytometry analysis. Patients were considered CD38 positive when expression was found in at least 20% of CLL cells.

### Fluorescence *in situ* hybrydization

Fluorescence *in situ* hybridization (FISH) method was used to determine cytogenetic abnormalities in leukemic cells. The locus-specific probes 17p13.1 (LSI TP53), 11q22.3 (LSI ATM), 13q14.3 (D13S319), 13q34, and the chromosome 12 centromere (Abott Diagnostics, USA) were used. Procedures were performed according to the manufacturer’s protocol. Probes were denatured at 73°C for 5 min and then applied to the designated areas of the slides. Following an overnight hybridization the slides were stained with DAPI. The analysis was performed using a BX51 fluorescence microscope (Olympus, USA), and CytoVision image analysis system. At least 200 nuclei were assessed for each probe, and the border value for positive result was 20%.

### Assessment of therapy outcome and survival time

In the group of subjects enrolled into the study the following clinical data were analyzed: time from diagnosis to starting of the therapy (time to progression-TTP), chemotherapy regimens ordered and chemotherapy outcome (overall response rate – ORR), probability of progression free survival (PFS) and probability of overall survival (OS). The criteria of therapy response proposed by WG- IWCLL in 2008 [49] based on WG- NCI criteria from 1996 [50] were used. The complete response required the absence of symptoms and organomegaly, normal complete cell counts of peripheral blood and less than 30% of lymphocytes in bone marrow for at least 2 months. When size of the lymph nodes, spleen and liver, together with the peripheral blood data, were at least 50% better than pre-treatment values, the partial response was achieved. Other patients were considered non-responders.

### Statistical analysis

Statistical analysis was performed with STATISTICA 12.0 software for Windows. The results were shown as median or mean values with standard deviation. The Mann–Whitney and Wilcoxon tests were used for groups comparison. The Kaplan–Meier method was employed to calculate the survival analysis. Multivariate analysis of independent clinical factors for survival was tested by linear regression. Value of *p <* 0.05 was considered to be statistically significant.

## Abbreviations

CLL: chronic lymphocytic leukemia
TTP: time to progression
PFS: progression free survival
OS: overall survival time
miR: micro RNA
MoAb: monoclonal antibody
IgVH: immunoglobulin heavy chain genes
PBMC: peripheral blood mononuclear cells
qRT-PCR: quantitative reverse transcriptase real-time PCR
cDNA: complementary DNA
Ct: cycle threshold
MACS: magnetic activated cell sorting
FISH: fluorescence *in situ* hybridization
